# Management of pneumothorax in the setting of chronic non-invasive positive pressure ventilation: A case series

**DOI:** 10.1016/j.rmcr.2022.101755

**Published:** 2022-10-27

**Authors:** Avnee J. Kumar, Alexander Hjelmaas, Benjamin H. Singer

**Affiliations:** Division of Pulmonary and Critical Care Medicine, Department of Internal Medicine, University of Michigan Medical School, Ann Arbor, MI, USA

**Keywords:** Pneumothorax, Non-invasive ventilation, Muscular dystrophy

## Abstract

There is a growing population of patients who require chronic noninvasive ventilation. While these patients often have no parenchymal lung disease, the use of positive pressure ventilation itself predisposes to both initial and recurrent pneumothoraces. Furthermore, generally accepted pneumothorax management strategies, such as removing a chest tube after liberation from positive pressure ventilation, are not possible in this population. Despite this, there is a lack of clear guidance on management of pneumothorax in the chronically ventilated patient. In this case series, we discuss the management of pneumothoraces in patients requiring chronic noninvasive mechanical ventilation in our Assisted Ventilation Clinic (AVC). Our experience suggests a potential role of definitive treatment of the initial pneumothorax to prevent reoccurrence.

## Abbreviations

AVC: Assisted Ventilation Clinic
PPV: Positive Pressure Ventilation

## 1 Introduction

Barotrauma, resulting in alveolar rupture and subsequent pneumothorax, is a potentially serious complication of positive pressure ventilation (PPV) in patients with neuromuscular respiratory failure. In patients with cervical spinal cord injury, the recommendation is to ventilate with higher tidal volumes if there is not a contraindication, which is a risk factor for barotrauma [1]. When pneumothorax occurs during acute PPV, there is a lower threshold for management with chest tube placement, with removal of the tube after liberation from mechanical ventilation [2]. For patients who require lifelong invasive or noninvasive mechanical ventilation (NIV) for chronic respiratory failure, appropriate management is unclear. The current literature regarding management of pneumothorax in chronic PPV is limited, and at least one case report highlights the possibility of mortality related to pneumothorax in a patient with Duchenne muscular dystrophy [3]. These limited data cause considerable distress among the families and uncertainty for the care team.

The Adult Assisted Ventilation Clinic (10.13039/501100005613AVC) at the 10.13039/100007270University of Michigan provides multidisciplinary care to patients with chronic neuromuscular respiratory failure utilizing chronic invasive and non-invasive ventilatory support. Here, we discuss six cases of initial pneumothorax that have occurred among patients of the UM AVC 2010–2019 (Table 1).

**Table 1:** Characteristics of six cases of pneumothorax in patients with chronic neuromuscular respiratory failure.

	Age/Gender	Neuromuscular Disease	Tidal Volume	Tracheostomy	Symptomatic?	Cough Assist Use	Reoccurrence	Initial Management	Secondary Management
Case 1	36M	C2 Spinal Cord Injury	950	Yes	Yes	Yes	Yes	Chest Tube	Pleurodesis
Case 2	19M	Central Core Myopathy	450	Yes	Yes	Yes	No	Chest Tube	None
Case 3	25M	Duchenne's Muscular Dystrophy	400	No	Yes	Yes	Yes	Chest Tube	VATS
Case 4	30M	Duchenne's Muscular Dystrophy	950	Yes	No	Yes	No	Chest Tube	None^a^
Case 5	46F	Fascioscapulohumeral muscular dystrophy	275	Yes	No	Yes	Yes	Chest Tube	None^a^
Case 6	21M	Duchenne's Muscular Dystrophy	615	No	No	Yes	Yes	Oxygen	VATS

a Died within 2 years of initial pneumothorax.

## 2 Case reports

The first patient, a 36-year-old male with 22-year history of C2 quadriplegia presented large left lateral pneumothorax. Prior to presentation, his ventilator settings were assist/control with a tidal volume of 900mL and a positive end expiratory pressure (PEEP) of 5 cm H_2_O in addition to cough assist. Left lateral 14 French tube thoracostomy was placed. Pneumothorax resolved and the patient was discharged to home and instructed not to use his cough assist. Three weeks later, follow up chest x-ray showed redevelopment of 2.2 cm left apical pneumothorax for which a left lateral 24 French chest tube was placed with resolution of pneumothorax. Bedside talc pleurodesis was performed. Course was complicated by pneumonia. Patient has been without pneumothorax recurrence since, however has been reluctant to use his cough assist.

The second case is a 19-year-old male with Central Core Myopathy who presented with a left sided pneumothorax after being bagged for a mucous plug at his school. Prior to presentation, his ventilator settings Pressure Control/Average Volume Assured Pressure Support (PC/AVAPS) mode with a tidal volume of 450 mL and a positive end expiratory pressure of 8 cm H_2_O, in addition to cough assist. Chest tube was placed with resolution of his pneumothorax, and he has had no reoccurrence since.

The third case is a 25-year-old male with Duchenne Muscular Dystrophy who presented with a large upper-left pneumothorax and a 3.1 cm left apical bleb. Prior to presentation, his ventilator settings PC/AVAPS mode with a tidal volume of 400 mL and a positive end expiratory pressure of 8 cm H_2_O, in addition to cough assist. A chest tube was placed with some, but not complete pneumothorax resolution. Pleurodesis was not performed given concern for lack of pleural apposition. Two weeks later, the patient presented with a pneumothorax, for which a chest tube was placed. Video-assisted Thoracoscopic Surgery (VATS) left blebectomy and additional tube thoracostomy placement was performed. Pleurodesis was not performed due to increased bleeding/friability of lung tissue. Patient has been without pneumothorax recurrence since VATS procedure.

Our fourth case is a 30-year-old male with Duchenne Muscular Dystrophy who presented with a left lateral pneumothorax. Prior to presentation, his ventilator settings were assist control with a tidal volume of 950 mL and a positive end expiratory pressure of 5 cm H_2_O, in addition to cough assist. A chest tube was placed with resolution of pneumothorax. He stopped using his cough assist. He died 1 year later of multiorgan failure and shock in the setting of an infected pleural space.

Our fifth case is a 46-year-old female with Facioscapulohumeral Muscular Dystrophy who presented with a left lateral pneumothorax during an admission for tracheostomy placement. Prior to presentation, her ventilator settings were assist control with a tidal volume of 275 mL and a positive end expiratory pressure of 5 cm H_2_O, in addition to cough assist. Chest tube was placed with resolution of pneumothorax, however, it reoccurred in the same admission, so another tube was placed with resolution. She improved and was discharged but died unexpectedly a week later.

Our sixth case is a 21-year-old male with Duchenne's Muscular Dystrophy who presented with a right sided pneumothorax. Prior to presentation, his ventilator settings were Intelligent Volume Assured Pressure Support (IVAPS) with a tidal volume of 615 mL and a positive end expiratory pressure of 7 cm H_2_O, in addition to cough assist. His 1 cm pneumothorax was at first managed conservatively with increased oxygen, however, it expanded, so a chest tube was placed and talc pleurodesis performed and he was discharged. Two days later, he was noted to have a pneumothorax, so was admitted, and had a chest tube followed by a VATS. He did develop a persistent hydropneumothorax which was managed with observation until his death 2 years later.

## 3 Discussion

Pneumothoraces have relatively high rate of recurrence, with estimates of recurrence ranging from 30 to 50% [3,4]. Pneumothoraces are stratified as primary or secondary, with the former being not associated with underlying pulmonary disorder. Despite the high rate of recurrence, seeking a more definitive preventative treatment is not typical for a primary pneumothorax due to the increased morbidity associated with these more invasive procedures [2]. There is equipoise regarding potential stratification of patients with primary pneumothorax. Some studies have suggested methods of risk-stratifying patients who present with primary spontaneous pneumothorax, with those at higher risk of recurrence receiving more definitive intervention rather than more conservative non-surgical treatment. One such study suggested use of a CT-based “lung dystrophy severity score” to guide management [5]. On the other hand, patients with secondary pneumothorax, defined as a pneumothorax with underlying lung disease, more aggressive up-front treatment to prevent recurrence may be warranted and lead to less morbidity in the long-term. The AVC has cared for between 400 to over 900 patients utilizing PPV during the ascertainment period of these cases, highlighting that pneumothorax in chronic PPV is a rare event.

These cases demonstrate the importance of exploring more definitive treatment to prevent recurrence of pneumothorax for patients who require chronic ventilation. Parenchymal lung disease, such as emphysema, is considered a clear etiology of secondary pneumothorax. However, there seems to exist an ambiguity in defining pneumothorax in the setting of chronic ventilation as primary or secondary in nature, and this ambiguity may lead to sub-optimal management. Out of our 6 cases, 3 (50%) developed reoccurrence despite no primary known parenchymal lung diseases. Out of the three cases that did not develop reoccurrence, one died shortly after initial admission. A second died a year later in the setting of an infected pleural space.

Previous case reports have emphasized the need for clinicians to have a higher suspicion for pneumothorax in patients using non invasive ventilation devices [3]. In half of our six cases, the patient did not have symptoms on initial presentation. Furthermore, recurrent pneumothoraces were often found via surveillance imaging, rather than being symptom guided.

Another prior case study pointed out a link between positive-pressure ventilation devices, namely those that utilize “air stacking” to facilitate pulmonary clearance, and incidence of pneumothorax. The report argues that patients with a known pulmonary pathology should avoid, or exercise caution, using cough assist and similar devices [6]. All six of our cases utilized cough assist devices. Practice varied in terms of temporary discontinuation of the cough assist after pneumothorax. In two cases, there was discontinuation of the device, but still a recurrent pneumothorax. Several of the patients discontinued their device due to concern for causing another pneumothorax. Last, one of our patients, who died two years after pneumothorax, had reportedly independently discontinued her device. She died of sepsis in the setting of an infected pleural space. Although we cannot directly relate these events, we note that discontinuation of cough assist devices can lead to poor pulmonary clearance and infections.

## 4 Conclusion

• These cases demonstrate the need for a stronger recommendation of definitive treatment to prevent recurrence of pneumothorax in chronically ventilated patients.
• Appropriate management of pneumothorax could lead to decreased hospital admissions and associated morbidities. One suggestion would be to include chronic NIPPV as a criterion of diagnosing secondary spontaneous pneumothorax to encourage treatment that will help prevent recurrence.
• Further study of pneumothorax incidence, recurrence, and overall morbidity in chronic NIPPV patients who receive only tube thoracostomy versus those receiving tube thoracostomy plus VATS, thoracotomy, or pleurodesis may be warranted.

## Declaration of competing interest

The authors have no conflicts of interest.Authors AJK, AH, BHS participated in collection, analysis and interpretation of data; in the writing of the manuscript; and in the decision to submit the manuscript for publication.
